# 2-Chloro-3-(4-methyl­anilino)naph­tha­lene-1,4-dione

**DOI:** 10.1107/S1600536812045229

**Published:** 2012-11-17

**Authors:** Li-Jiu Gao, Yun Liu

**Affiliations:** a Department of Chemistry and Chemical Engineering, Xuzhou Normal University, Xuzhou, Jiangsu 221116, People’s Republic of China

## Abstract

In the title compound, C_17_H_12_ClNO_2_, the naphtho­quinone system is essentially planar [maximum deviation = 0.078 (2) Å] and makes a dihedral angle of 52.38 (7)° with the benzene ring. The crystal structure features N—H⋯O inter­actions.

## Related literature

For the properties of substituted naphtho­quinones, see: Batton *et al.* (2000 ▶); Monks *et al.* (1992 ▶). For standard bond lengths, see: Allen *et al.* (1987 ▶). For the structure of 2-hy­droxy­quinoxaline, see: Stępień *et al.* (1976 ▶).
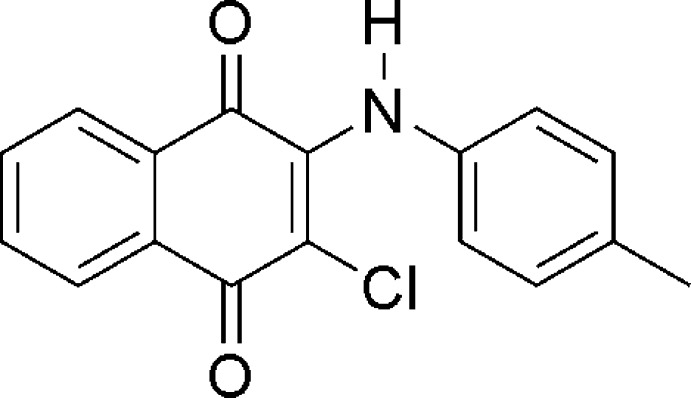


## Experimental

### 

#### Crystal data

C_17_H_12_ClNO_2_
*M* *_r_* = 297.73
Orthorhombic, 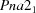

*a* = 12.1614 (10) Å
*b* = 22.4915 (18) Å
*c* = 5.0444 (4) Å
*V* = 1379.79 (19) Å^3^
*Z* = 4
Mo *K*α radiation
μ = 0.28 mm^−1^
*T* = 296 K
0.2 × 0.2 × 0.1 mm

#### Data collection

Enraf–Nonius CAD-4 diffractometer
Absorption correction: ψ scan (*XCAD4*; Harms & Wocadlo, 1995 ▶) *T*_min_ = 0.946, *T*_max_ = 0.972
15471 measured reflections
2479 independent reflections
2420 reflections with *I* > 2σ(*I*)
*R*_int_ = 0.036
3 standard reflections every 200 reflections intensity decay: none

#### Refinement

*R*[*F* ^2^ > 2σ(*F* ^2^)] = 0.040
*wR*(*F* ^2^) = 0.119
*S* = 1.27
2479 reflections
195 parameters
1 restraint
H atoms treated by a mixture of independent and constrained refinement
Δρ_max_ = 0.41 e Å^−3^
Δρ_min_ = −0.42 e Å^−3^

### 

Data collection: *CAD-4 Software* (Enraf–Nonius, 1989 ▶); cell refinement: *CAD-4 Software*; data reduction: *XCAD4* (Harms & Wocadlo, 1995 ▶); program(s) used to solve structure: *SHELXS97* (Sheldrick, 2008 ▶); program(s) used to refine structure: *SHELXL97* (Sheldrick, 2008 ▶); molecular graphics: *SHELXTL* (Sheldrick, 2008 ▶); software used to prepare material for publication: *SHELXTL* and *PLATON* (Spek, 2009 ▶).

## Supplementary Material

Crystal structure: contains datablock(s) I, global. DOI: 10.1107/S1600536812045229/ds2213sup1.cif

Structure factors: contains datablock(s) I. DOI: 10.1107/S1600536812045229/ds2213Isup2.hkl

Supplementary material file. DOI: 10.1107/S1600536812045229/ds2213Isup3.cml

Additional supplementary materials:  crystallographic information; 3D view; checkCIF report

## Figures and Tables

**Table 1:** Hydrogen-bond geometry (Å, °)

*D*—H⋯*A*	*D*—H	H⋯*A*	*D*⋯*A*	*D*—H⋯*A*
N—H⋯O2	0.70 (2)	2.23 (3)	2.611 (3)	116 (2)

**Fig. 1.**
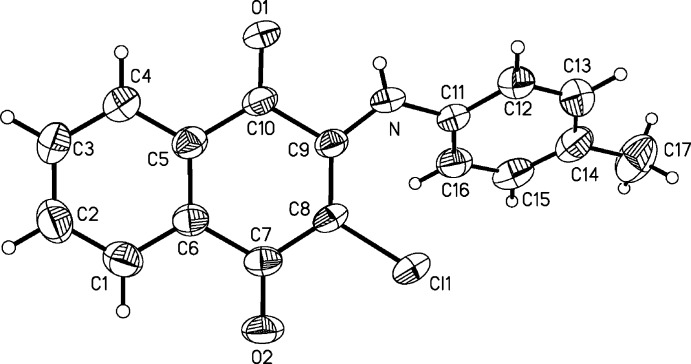
Ellipsoid plot.

**Fig. 2.**
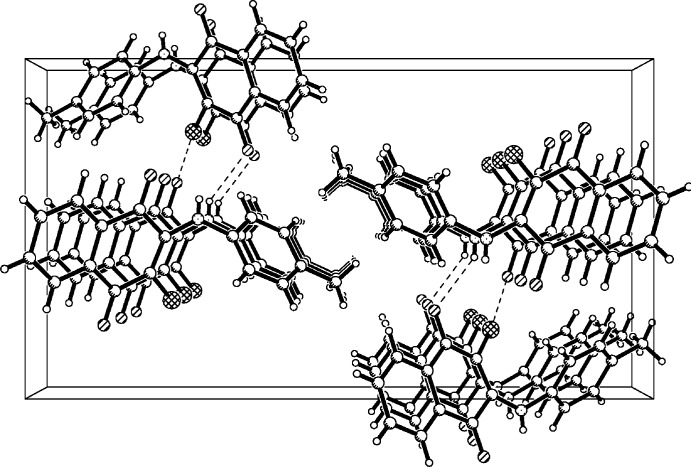
Packing diagram.

## supplementary crystallographic information

### Comment

The substituted naphthoquinone have a diversity of biological activity and are playing an increasingly important role in developing new pharmaceuticals [Batton *et al.*, 2000; Monks *et al.*, 1992]. In our ongoing research work on the syntheses of amino-substituted naphthoquinones, we have prepared the title compound, (I), as one of the products. As part of this study, we have undertaken an X-ray crystallographic analysis of (I) in order to confirm its structure.

In the title compound, the bond lengths and angles of the title molecule (Fig. 1) are within normal ranges (Allen *et al.*, 1987). The naphthoquinone ring[C1—C10] is essentially planar. The naphthoquinone ring makes the dihedral angle 52.38 (0.07) with the benzene ring [C11—C16]. Although atoms C9 and C11 attached to atom N are all of *sp*^2^ hybridization, their different environments cause slight differences in the N—C9, N—C11 bond lengths, and in the C9—N—H, C11—N—H angles (Table 1). The molecular packing is stabilized by intermolecular N—H···O hydrogen bonds (Table 2).

### Experimental

To a stirred solution of naphthoquinone (1.0 eq) in 10 ml of acetonitrile, potassium carbonate (3.0 eq) was added. The mixture was stirred at room temperature for 5 min, followed by the addition of aniline (1.0 eq) and silver nitrate (0.1 mmol). The reaction mixture was refluxed for 10 h until complete consumption of starting material was observed on TLC. The reaction mixture was purified over silica gel (EtOAc/hexane)to afford the product in 96% yield.

### Refinement

The H atoms were geometrically placed and were treated as riding, with C—H = 0.93 Å.

### Crystal data

**Table:**

C_17_H_12_ClNO_2_	*D*_x_ = 1.433 Mg m^−^^3^
*M**_r_* = 297.73	Melting point: 475 K
Orthorhombic, *P**n**a*2_1_	Mo *K*α radiation, λ = 0.71073 Å
Hall symbol: P 2c -2n	Cell parameters from 25 reflections
*a* = 12.1614 (10) Å	θ = 9–12°
*b* = 22.4915 (18) Å	µ = 0.28 mm^−^^1^
*c* = 5.0444 (4) Å	*T* = 296 K
*V* = 1379.79 (19) Å^3^	Block, red
*Z* = 4	0.2 × 0.2 × 0.1 mm
*F*(000) = 616	

### Data collection

**Table:**

Enraf–Nonius CAD-4 diffractometer	2420 reflections with *I* > 2σ(*I*)
Radiation source: fine-focus sealed tube	*R*_int_ = 0.036
Graphite monochromator	θ_max_ = 25.4°, θ_min_ = 1.8°
ω/2θ scans	*h* = −14→14
Absorption correction: ψ scan (*XCAD4*; Harms & Wocadlo, 1995)	*k* = −26→27
*T*_min_ = 0.946, *T*_max_ = 0.972	*l* = −6→6
15471 measured reflections	3 standard reflections every 200 reflections
2479 independent reflections	intensity decay: none

### Refinement

**Table:**

Refinement on *F*^2^	Secondary atom site location: difference Fourier map
Least-squares matrix: full	Hydrogen site location: inferred from neighbouring sites
*R*[*F*^2^ > 2σ(*F*^2^)] = 0.040	H atoms treated by a mixture of independent and constrained refinement
*wR*(*F*^2^) = 0.119	*w* = 1/[σ^2^(*F*_o_^2^) + (0.0725*P*)^2^ + 0.1832*P*] where *P* = (*F*_o_^2^ + 2*F*_c_^2^)/3
*S* = 1.27	(Δ/σ)_max_ = 0.001
2479 reflections	Δρ_max_ = 0.41 e Å^−^^3^
195 parameters	Δρ_min_ = −0.42 e Å^−^^3^
1 restraint	Extinction correction: *SHELXL97* (Sheldrick, 2008), Fc^*^=kFc[1+0.001xFc^2^λ^3^/sin(2θ)]^-1/4^
Primary atom site location: structure-invariant direct methods	Extinction coefficient: 0.083 (7)

### Special details

**Table:**

Geometry. All e.s.d.'s (except the e.s.d. in the dihedral angle between two l.s. planes) are estimated using the full covariance matrix. The cell e.s.d.'s are taken into account individually in the estimation of e.s.d.'s in distances, angles and torsion angles; correlations between e.s.d.'s in cell parameters are only used when they are defined by crystal symmetry. An approximate (isotropic) treatment of cell e.s.d.'s is used for estimating e.s.d.'s involving l.s. planes.
Refinement. Refinement of *F*^2^ against ALL reflections. The weighted *R*-factor *wR* and goodness of fit *S* are based on *F*^2^, conventional *R*-factors *R* are based on *F*, with *F* set to zero for negative *F*^2^. The threshold expression of *F*^2^ > σ(*F*^2^) is used only for calculating *R*-factors(gt) *etc*. and is not relevant to the choice of reflections for refinement. *R*-factors based on *F*^2^ are statistically about twice as large as those based on *F*, and *R*- factors based on ALL data will be even larger. Flack parameter does not have any meaning here. Obviously anomalous contribution from Cl was not good enough to resolve the chirality.

### Fractional atomic coordinates and isotropic or equivalent isotropic displacement parameters (Å^2^)

**Table:**

	*x*	*y*	*z*	*U*_iso_*/*U*_eq_	
Cl1	0.79748 (5)	0.74205 (2)	0.55999 (16)	0.0433 (2)	
C10	1.05237 (17)	0.70460 (10)	0.0576 (6)	0.0407 (5)	
C8	0.87671 (18)	0.71478 (10)	0.3028 (5)	0.0351 (5)	
O1	0.73453 (13)	0.64787 (8)	0.1976 (5)	0.0514 (5)	
C9	0.97837 (19)	0.73816 (10)	0.2467 (5)	0.0358 (5)	
N	1.02703 (18)	0.78611 (10)	0.3494 (5)	0.0453 (6)	
O2	1.14579 (14)	0.72164 (10)	0.0280 (5)	0.0623 (6)	
C6	0.89985 (18)	0.63439 (10)	−0.0415 (5)	0.0378 (5)	
C11	0.97909 (19)	0.83701 (11)	0.4684 (5)	0.0393 (5)	
C7	0.82917 (18)	0.66522 (9)	0.1601 (5)	0.0376 (5)	
C5	1.00808 (18)	0.65242 (10)	−0.0834 (5)	0.0395 (5)	
C1	0.8574 (2)	0.58704 (11)	−0.1871 (6)	0.0497 (7)	
H1A	0.7848	0.5752	−0.1626	0.060*	
C12	1.0318 (2)	0.86289 (12)	0.6821 (6)	0.0480 (6)	
H12A	1.0960	0.8463	0.7492	0.058*	
C16	0.8851 (2)	0.86286 (12)	0.3654 (6)	0.0475 (6)	
H16A	0.8502	0.8463	0.2191	0.057*	
C3	1.0317 (2)	0.57524 (13)	−0.4066 (7)	0.0586 (7)	
H3A	1.0758	0.5551	−0.5276	0.070*	
C4	1.0739 (2)	0.62259 (13)	−0.2648 (6)	0.0522 (7)	
H4A	1.1464	0.6345	−0.2908	0.063*	
C15	0.8440 (2)	0.91351 (12)	0.4826 (7)	0.0522 (7)	
H15A	0.7807	0.9306	0.4127	0.063*	
C13	0.9881 (2)	0.91369 (14)	0.7954 (7)	0.0579 (8)	
H13A	1.0236	0.9307	0.9400	0.069*	
C14	0.8930 (3)	0.93989 (12)	0.6993 (7)	0.0572 (7)	
C2	0.9233 (3)	0.55771 (12)	−0.3683 (7)	0.0582 (8)	
H2A	0.8949	0.5260	−0.4651	0.070*	
C17	0.8458 (3)	0.99422 (15)	0.8318 (9)	0.0829 (12)	
H17A	0.7804	1.0064	0.7402	0.124*	
H17B	0.8988	1.0258	0.8269	0.124*	
H17C	0.8281	0.9851	1.0128	0.124*	
H	1.082 (2)	0.7883 (12)	0.317 (6)	0.033 (7)*	

### Atomic displacement parameters (Å^2^)

**Table:**

	*U*^11^	*U*^22^	*U*^33^	*U*^12^	*U*^13^	*U*^23^
Cl1	0.0329 (3)	0.0546 (3)	0.0424 (3)	0.00269 (19)	0.0127 (3)	0.0028 (3)
C10	0.0276 (11)	0.0539 (11)	0.0407 (12)	−0.0004 (8)	0.0058 (10)	−0.0035 (12)
C8	0.0240 (10)	0.0460 (12)	0.0351 (11)	0.0060 (8)	0.0057 (9)	0.0038 (9)
O1	0.0278 (8)	0.0552 (10)	0.0712 (13)	−0.0061 (7)	0.0072 (9)	−0.0009 (9)
C9	0.0259 (11)	0.0456 (11)	0.0357 (12)	0.0016 (8)	0.0017 (10)	−0.0012 (9)
N	0.0226 (10)	0.0570 (13)	0.0564 (14)	−0.0033 (9)	0.0085 (10)	−0.0117 (10)
O2	0.0310 (9)	0.0820 (13)	0.0739 (15)	−0.0127 (8)	0.0194 (10)	−0.0292 (12)
C6	0.0329 (12)	0.0402 (10)	0.0402 (12)	0.0013 (9)	−0.0011 (9)	0.0046 (9)
C11	0.0294 (10)	0.0460 (11)	0.0425 (13)	−0.0028 (9)	0.0077 (10)	−0.0030 (10)
C7	0.0285 (10)	0.0397 (11)	0.0446 (13)	0.0014 (9)	0.0001 (9)	0.0085 (9)
C5	0.0321 (11)	0.0449 (11)	0.0414 (13)	0.0044 (9)	−0.0002 (10)	0.0000 (10)
C1	0.0414 (13)	0.0452 (13)	0.0626 (17)	−0.0036 (11)	−0.0045 (12)	0.0005 (12)
C12	0.0369 (13)	0.0566 (14)	0.0504 (15)	0.0004 (10)	0.0004 (12)	−0.0033 (12)
C16	0.0359 (12)	0.0593 (15)	0.0471 (14)	−0.0015 (11)	0.0023 (11)	0.0026 (12)
C3	0.0538 (15)	0.0609 (15)	0.0612 (18)	0.0056 (11)	0.0077 (15)	−0.0217 (15)
C4	0.0399 (13)	0.0591 (14)	0.0575 (17)	0.0010 (11)	0.0054 (13)	−0.0110 (13)
C15	0.0394 (13)	0.0534 (14)	0.0638 (19)	0.0082 (11)	0.0123 (13)	0.0158 (12)
C13	0.0569 (17)	0.0587 (16)	0.0581 (18)	−0.0068 (13)	0.0048 (15)	−0.0135 (13)
C14	0.0584 (17)	0.0481 (13)	0.0650 (19)	0.0006 (12)	0.0234 (15)	−0.0004 (13)
C2	0.0606 (17)	0.0498 (13)	0.0640 (19)	0.0001 (12)	−0.0094 (14)	−0.0143 (12)
C17	0.101 (3)	0.0615 (18)	0.086 (3)	0.0188 (19)	0.032 (2)	−0.0042 (18)

### Geometric parameters (Å, º)

**Table:**

Cl1—C8	1.728 (2)	C12—C13	1.383 (4)
C10—O2	1.208 (3)	C12—H12A	0.9300
C10—C5	1.474 (3)	C16—C15	1.377 (4)
C10—C9	1.513 (3)	C16—H16A	0.9300
C8—C9	1.373 (3)	C3—C4	1.382 (4)
C8—C7	1.447 (3)	C3—C2	1.389 (4)
O1—C7	1.230 (3)	C3—H3A	0.9300
C9—N	1.335 (3)	C4—H4A	0.9300
N—C11	1.418 (3)	C15—C14	1.379 (5)
N—H	0.69 (3)	C15—H15A	0.9300
C6—C1	1.393 (4)	C13—C14	1.386 (5)
C6—C5	1.393 (3)	C13—H13A	0.9300
C6—C7	1.501 (3)	C14—C17	1.506 (4)
C11—C12	1.382 (4)	C2—H2A	0.9300
C11—C16	1.383 (4)	C17—H17A	0.9600
C5—C4	1.389 (4)	C17—H17B	0.9600
C1—C2	1.383 (4)	C17—H17C	0.9600
C1—H1A	0.9300		
			
O2—C10—C5	122.5 (2)	C13—C12—H12A	120.3
O2—C10—C9	118.6 (2)	C15—C16—C11	119.1 (3)
C5—C10—C9	118.94 (18)	C15—C16—H16A	120.4
C9—C8—C7	123.5 (2)	C11—C16—H16A	120.4
C9—C8—Cl1	121.39 (19)	C4—C3—C2	119.9 (3)
C7—C8—Cl1	115.08 (16)	C4—C3—H3A	120.0
N—C9—C8	129.0 (2)	C2—C3—H3A	120.0
N—C9—C10	112.6 (2)	C3—C4—C5	119.9 (3)
C8—C9—C10	118.3 (2)	C3—C4—H4A	120.0
C9—N—C11	129.4 (2)	C5—C4—H4A	120.0
C9—N—H	113 (2)	C16—C15—C14	122.6 (3)
C11—N—H	116 (2)	C16—C15—H15A	118.7
C1—C6—C5	119.5 (2)	C14—C15—H15A	118.7
C1—C6—C7	119.9 (2)	C12—C13—C14	121.8 (3)
C5—C6—C7	120.6 (2)	C12—C13—H13A	119.1
C12—C11—C16	119.9 (2)	C14—C13—H13A	119.1
C12—C11—N	118.6 (2)	C15—C14—C13	117.1 (3)
C16—C11—N	121.3 (2)	C15—C14—C17	122.4 (3)
O1—C7—C8	122.8 (2)	C13—C14—C17	120.5 (3)
O1—C7—C6	119.6 (2)	C1—C2—C3	120.4 (3)
C8—C7—C6	117.64 (19)	C1—C2—H2A	119.8
C4—C5—C6	120.3 (2)	C3—C2—H2A	119.8
C4—C5—C10	119.5 (2)	C14—C17—H17A	109.5
C6—C5—C10	120.2 (2)	C14—C17—H17B	109.5
C2—C1—C6	119.9 (2)	H17A—C17—H17B	109.5
C2—C1—H1A	120.1	C14—C17—H17C	109.5
C6—C1—H1A	120.1	H17A—C17—H17C	109.5
C11—C12—C13	119.5 (3)	H17B—C17—H17C	109.5
C11—C12—H12A	120.3		

### Hydrogen-bond geometry (Å, º)

**Table:**

*D*—H···*A*	*D*—H	H···*A*	*D*···*A*	*D*—H···*A*
N—H···O2	0.70 (2)	2.23 (3)	2.611 (3)	116 (2)
